# Young coconut juice can accelerate the healing process of cutaneous wounds

**DOI:** 10.1186/1472-6882-12-252

**Published:** 2012-12-12

**Authors:** Nisaudah Radenahmad, Farid Saleh, Ibrahim Sayoh, Kitja Sawangjaroen, Patchara Subhadhirasakul, Piyakorn Boonyoung, Wilart Rundorn, Winyou Mitranun

**Affiliations:** 1 Department of Anatomy, Prince of Songkla University, Hat Yai, 90112, Thailand; 2 Department of Anatomy, College of Medicine, Al-Imam Muhammad Ibn Saud Islamic University, Riyadh, 13317-4233, Kingdom of Saudi Arabia; 3 Faculty of Science and Technology, Princess of Naradhiwas University, Narathiwat, 96000, Thailand; 4 Department of Pharmacology, Faculty of Science, Prince of Songkla University, Hat Yai, 90112, Thailand; 5 Department of Pathology, Faculty of Medicine, Prince of Songkla University, Hat Yai, 90112, Thailand

**Keywords:** Young coconut juice, Wound healing, Estrogen, ER-α, ER-β, Ovariectomy

## Abstract

**Background:**

Estrogen has been reported to accelerate cutaneous wound healing. This research studies the effect of young coconut juice (YCJ), presumably containing estrogen-like substances, on cutaneous wound healing in ovairectomized rats.

**Methods:**

Four groups of female rats (6 in each group) were included in this study. These included sham-operated, ovariectomized (ovx), ovx receiving estradiol benzoate (EB) injections intraperitoneally, and ovx receiving YCJ orally. Two equidistant 1-cm full-thickness skin incisional wounds were made two weeks after ovariectomy. The rats were sacrificed at the end of the third and the fourth week of the study, and their serum estradiol (E2) level was measured by chemiluminescent immunoassay. The skin was excised and examined in histological sections stained with H&E, and immunostained using anti-estrogen receptor (ER-α an ER-β) antibodies.

**Results:**

Wound healing was accelerated in ovx rats receiving YCJ, as compared to controls. This was associated with significantly higher density of immunostaining for ER-α an ER-β in keratinocytes, fibroblasts, white blood cells, fat cells, sebaceous gland, skeletal muscles, and hair shafts and follicles. This was also associated with thicker epidermis and dermis, but with thinner hypodermis. In addition, the number and size of immunoreactive hair follicles for both ER-α and ER-β were the highest in the ovx+YCJ group, as compared to the ovx+EB group.

**Conclusions:**

This study demonstrates that YCJ has estrogen-like characteristics, which in turn seem to have beneficial effects on cutaneous wound healing.

## Background

One of the challenges of the current and coming centuries is the aging population. Age-related diseases like vascular and metabolic diseases often result in poor peripheral circulation leading to delayed wound healing, which in turn could lead to serious conditions like amputation.

In Thailand, and like the rest of the world, this has resulted in a significant burden on the government and other health care providers, which was estimated to cost millions of Baht per year. Considering the high prevalence and the extended time frame for treatment of established ulcers, there seems to be a distinct lack of effective management modalities for chronic wounds [1]. To date, there are no commercially-available drug treatments that specifically address delayed wound healing in the elderly.

Sex steroid hormones seem to have a profound influence on regulating skin maintenance and turnover. Estrogen maintains dermal thickness, and promotes both the maintenance of the extracellular matrix collagen levels and the structural integrity of the skin. In postmenopausal women, estrogen reduction causes deleterious skin changes, resulting in delayed cutaneous healing that is associated with increased inflammation, reduced matrix deposition, and unregulated protease activity [2]. Exogenous estrogen treatment reverses this delayed healing by reducing inflammation, and stimulating the deposition of matrix collagen and re-epithelialization [3,4]. Several studies have reported that hormone replacement therapy (HRT) may prevent the development of chronic wounds in postmenopausal women [5,6]. However, chronic HRT has been implicated with increased risk of heart disease, breast cancer, and stroke, and, accordingly, recommendations in relation to situations where the use of HRT would be highly beneficial remain unclear [7]. This indicates that there is a need for evaluation of alternatives to HRT, such as selective estrogen receptor modulators (SERMs).

Young coconut juice (YCJ) has been used in Thailand by menopausal women for decades to alleviate some symptoms associated with the decrease in the level of estrogen. We have recently shown that YCJ possesses *some* estrogenic properties, which were able to significantly reverse some pathologies associated with hormonal imbalance [8,9]. In this study, we investigated the possible beneficial effects of intake of YCJ on accelerating wound healing in ovx rats, a model often used for menopause. In addition, we examined the ability of YCJ to act as SERM, by examining the relative importance of estrogen receptors alpha and beta (ER-α and ER-β) in the possible acceleration of wound healing following YCJ administration.

## Materials and methods

### Plant material

Young coconut juice (*Cocos nucifera* L., Arecaceae) was collected from Tungngai district, Hat Yai, Songkhla, Thailand. It was then authenticated by Associate Professor Dr. Sanan Subhadhirasakul at the Department of Pharmacognosy and Pharmaceutical Botany, Faculty of Pharmaceutical Sciences, Prince of Songkla University, Hat Yai, Thailand. YCJ was dried, and the powder form was kept at -30°C until used. It was freshly reconstituted and prepared for oral intake every day. A complete description of YCJ, including its preparation and administration, is reported in our previous publications [8,9].

### Animals

All animals used were adult two-month old female Wistar rats weighing approximately 230 g. The animals were housed in a controlled environment at 25±1°C on an illumination schedule of 12 h light/12 h dark cycle. Rats had unrestricted access to standard pellet food and water. The study was approved by the Prince of Songkla University Animal Care and Use Ethics Committee, and was carried out in accordance with the Guiding Principles for the Care and Use of Research Animals set by this Committee.

### Experimental design

There were four groups (6 rats per group) included in this study. The first group consisted of ovx rats, the second group included sham-operated rats, the third group consisted of ovx rats injected intraperitoneally with exogenous estrogen (2.5 μg/kgBW of estradiol benzoate, EB) once a day, and the fourth group included ovx rats that received YCJ (100 mL/kgBW/day). The third and fourth groups were treated for one week (7 days treatment) and two weeks (14 days treatment). The dose of EB and YCJ in this study was based on the one reported in our earlier study, and in which dose standardization and optimal administration were set [8-10]. In this study, the administration of EB and YCJ was started two week after overiectomy was performed. Rats belonging to the first and second groups received deionized water instead of EB and YCJ. Two weeks following sham-operation or ovariectomy, all animals were wounded by making an incision at the dorsal surface 1cm below the scapula, which was 1.5 cm long and 3 mm deep (from the skin and panniculus carnosus muscle). The incision was left to heal by secondary intention (i.e. the wound edges were not closed by sutures). At the end of the experimental one week and two week period, all rats were sacrificed, and the whole initial wound area was biopsied and processed for light and transmission electron microscopy (TEM) analysis.

### Immunohistochemistry

Some of the biopsies were processed into paraffin blocks, and ten 5μm sections were collected from each block and mounted on 3-aminopropyltriethoxysilane (TESPA; Sigma)-coated slides. The first two sections were stained with H&E, and were used for light microscopy examination and anatomical orientation. Three of the remaining eight sections were immunostained with anti-ER-α monoclonal antibody (1:400 dilution; MAB447, Chemicon international, Temecula, CA, USA). Another three sections were immunostained with anti- ER-β polyclonal antibody (1:200 dilution; AB1410, Chemicon international, Temecula, CA, USA). The remaining two sections were used as negative controls. The immunostaining technique was described previously [8,9]. Negative controls included sections in which incubation with the primary antibody was replaced by tris phosphate buffer (TBS). Positive controls were sections from the uterus and ovary known to be positive for ER-α, and ER-β, respectively.

### Microscopic analysis by quantitative histomorphometry

Counting of all parameters was performed by two independent observers. An eye piece micrometer was mounted on a light microscope, and counting was made at x400 magnification. Readings from both observers were then added and the average was determined. Three areas of wound depth were determined and averaged from the perpendicular epidermis to the deepest part of the granulation tissue. Three areas of wound width: the top, the middle, and the bottom of wound were measured and averaged. The epidermal thickness was defined as the distance between the stratum granulosum and the stratum basale. The distance between the stratum basale and the upper part of hypodermis was measured for assessing the dermal thickness. Hypodermal thickness was measured as the distance between the bottom line of the dermis and the muscularis adiposa. Each reported thickness is an average of 25 measurements. Hair follicles were detected from the largest size of 10 hair follicle diameters in the dermis and hypodermis, averaged, and compared between each group. Image analysis and quantification of all parameters were performed using an Image Pro Plus program (DP11, Olympus SZX 12, Japan). Data was expressed as numbers per μm^2^. Mean ± SEM was used to compare the four groups.

### Serum estradiol

All the rats were sacrificed on the first day of the fourth and the fifth week, and their serum was collected and measured for estradiol (E2) using the chemiluminescent immunoassay (CIA) technique (ECLIA, Modular E 170C, Estradiol II 03000079 122, Roche, Germany).

### Statistical analysis

Statistical analysis was performed using the Kruskal-Wallis and the Mann-Whitney U-tests. Results were reported as mean ± SEM. P < 0.05 was considered significant. Correlation analysis between hair follicle diameter in the dermis and hypodermis and serum E2 level was performed using Spearman's rank correlation coefficient test.

## Results

### E2 serum level

**Figure 1:**
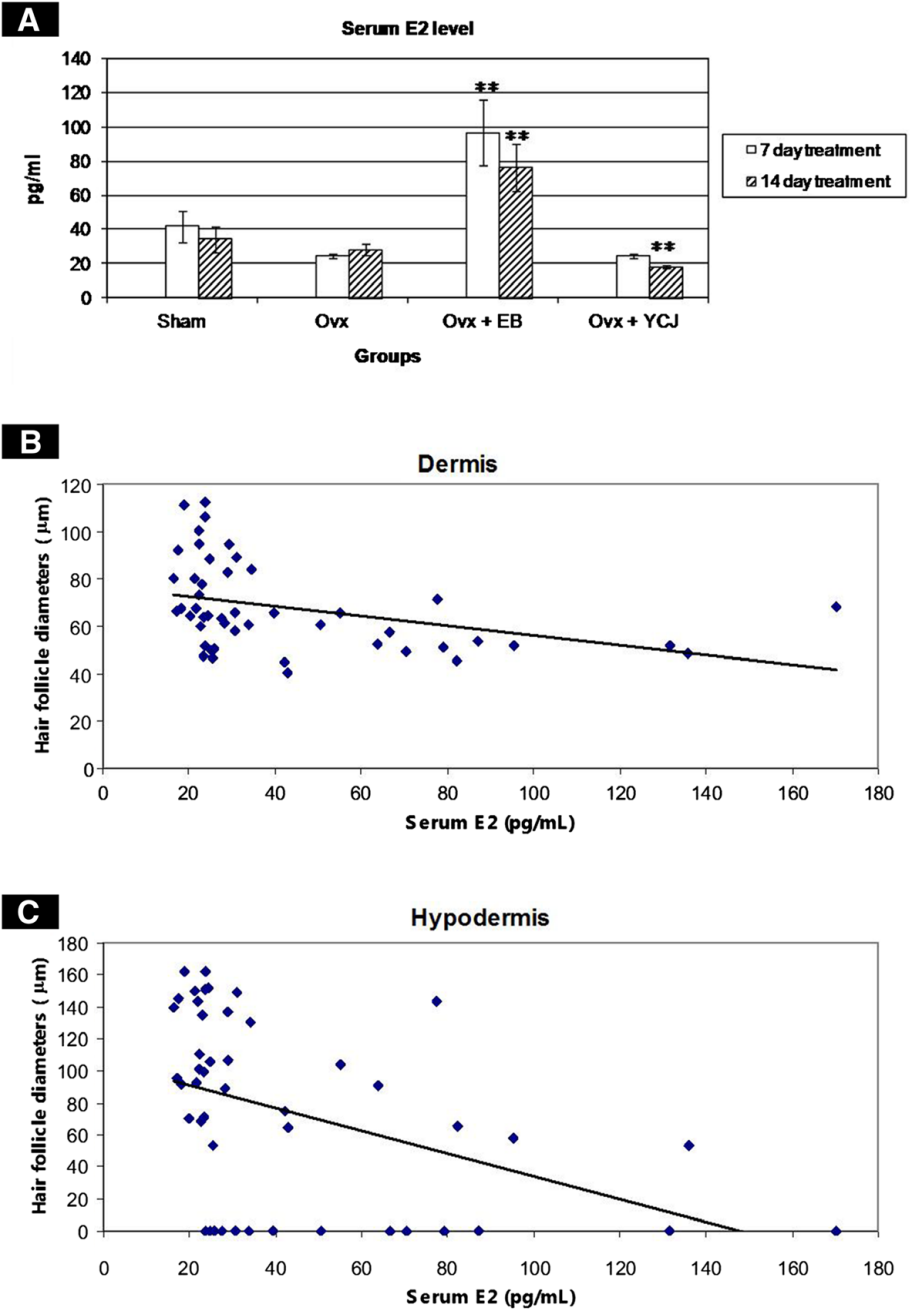
**A.Serum estradiol** (**E2**) **level** (**mean** ± **SEM**) **in the four groups examined.** Ovx = ovariectomized group; Ovx + EB = ovariectomized group receiving estradiol benzoate; Ovx + YCJ = ovariectomized group receiving young coconut juice. n = 6 in each group. ** P < 0.01 compared with ovx group. **B**. Negative correlation between the diameter of the hair follicles in the dermis, and serum E2 level in the four groups examined. **C**. Negative correlation between the diameter of the hair follicles in the hypodermis and serum E2 level in the four groups examined.

Our results showed that the circulating level of E2 was significantly (p < 0.01) less in the ovx+YCJ group following 14 days intake, as compared to the other groups. Such a trend was also seen following seven days intake, except when compared with the ovx group (Figure 1A). Using the diameter of the hair follicles both in the dermis and hypodermis as one of the parameters indicated in wound healing, we have noticed that there was a negative correlation between serum E2 level and hair follicle diameter following both seven and 14 days treatment (Figures 1B, C).

### Wound healing

#### General observation

Gross examination of the skin clearly showed that all wounds (ovx wounds and excision ones) of the ovx +YCJ group had significantly (p < 0.01) the fastest healing rate, which was associated with less redness, swelling, and exudates, as compared to the other groups (photos are presented as supplementary data with this manuscript (Additional file 1: Figure S1) and (Additional file 2: Figure S2). Such results were further confirmed by the histological examinations of the wounds, as described below.

#### Histological and immunohistochemical analyses

**Figure 2:**
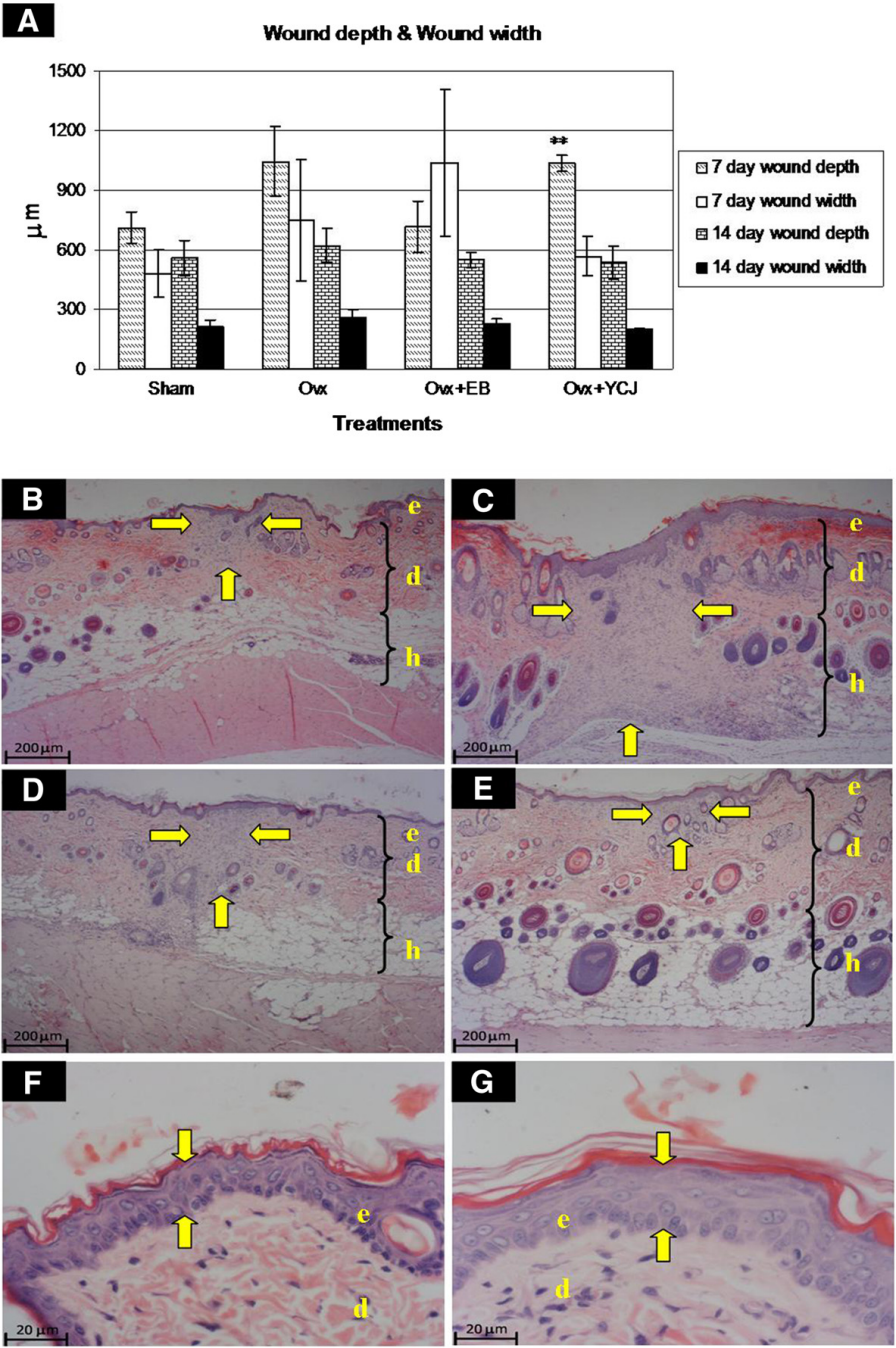
**A.Comparison of wound depth and width in the four groups.** Ovx = ovariectomized group; Ovx + EB = ovariectomized group receiving estradiol benzoate; Ovx + YCJ = ovariectomized group receiving young coconut juice. n = 6 in each group. ** P < 0.01 compared with the sham group. **B**-**E**. Sections from the dorsal skin wound areas following a 14-day-treatment period. (H&E; x4). The wound area is indicated by the yellow arrows. The wound area was the smallest in the Ovx+YCJ group **(E),** and the largest in the ovx group **(C).** Note the higher number and the bigger size of the hair follicles in the Ovx+YCJ group in both the dermis and hypodermis. ***B*** = Sham-operated group; ***C*** = Ovx group; ***D*** = Ovx+EB group; ***E*** = Ovx+YCJ group. ***e*** = epidermis; ***d*** = dermis; ***h*** = hypodermis. **F-G.** Epidermal thickness is indicated by the yellow arrows. The epidermis was the thinnest in the ovx group **(F),** and the thickest in the Ovx+YCJ group **(G).** Note that the collagen fibers (pink staining) in the dermis are much smaller and less in number in the ovx group, as compared to the Ovx+YCJ group. (H&E; x40).

**Figure 3:**
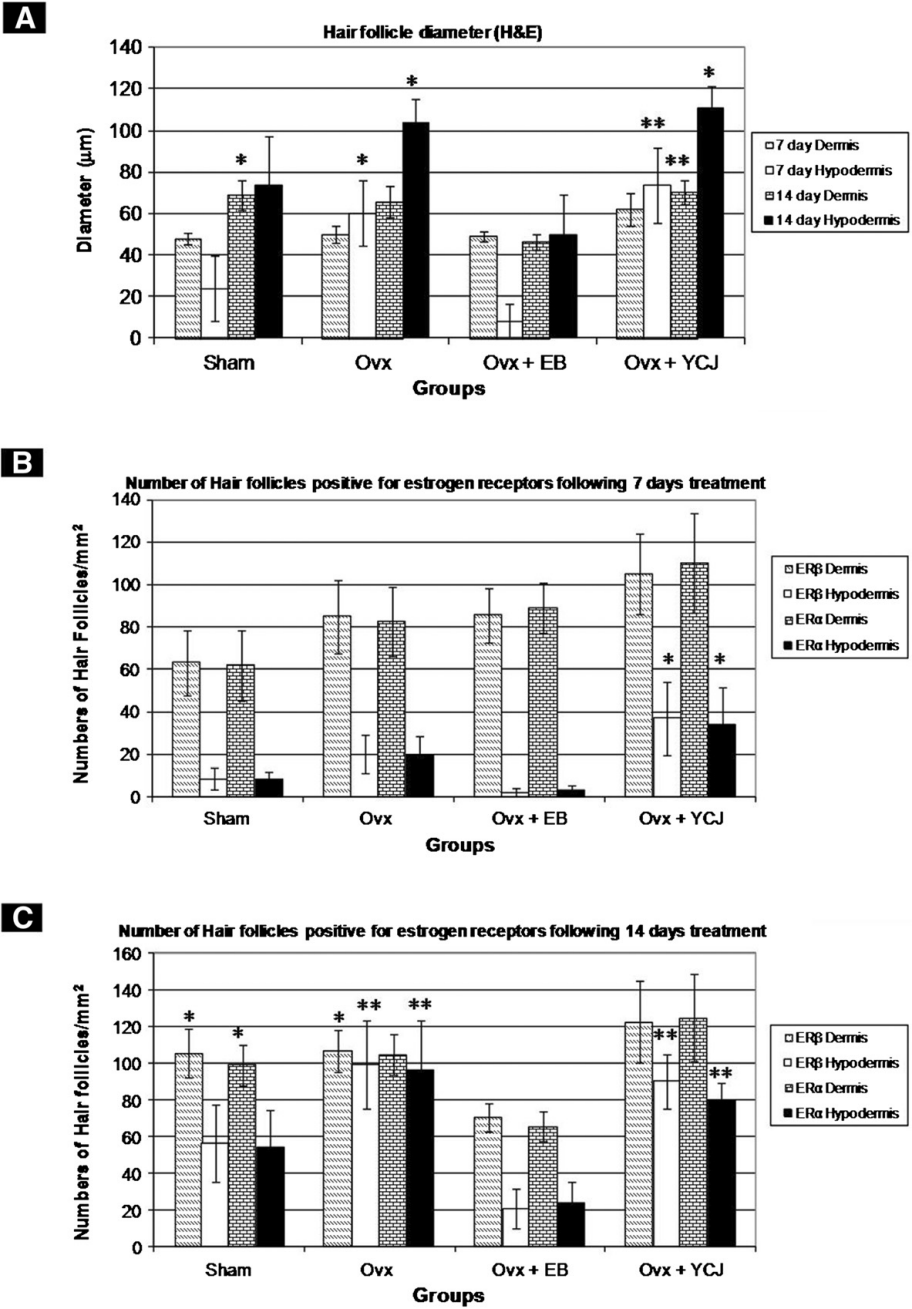
**A. Diameter of dermal and hypodermal hair follicles in the dorsal skin wound areas of the various groups.** The largest diameter was observed in the group receiving YCJ. **B, C.** Number of hair follicles positively-immunostained for ER-α, and ER-β, respectively. The number of these follicles was the highest in the group receiving YCJ. *Ovx* = ovariectomized group; *Ovx + EB* = ovariectomized group receiving estradiol benzoate; *Ovx + YCJ* = ovariectomized group receiving young coconut juice. n = 6 in each group. * P < 0.05, and **p < 0.01 compared with the ovx+EB group, respectively.

**Figure 4:**
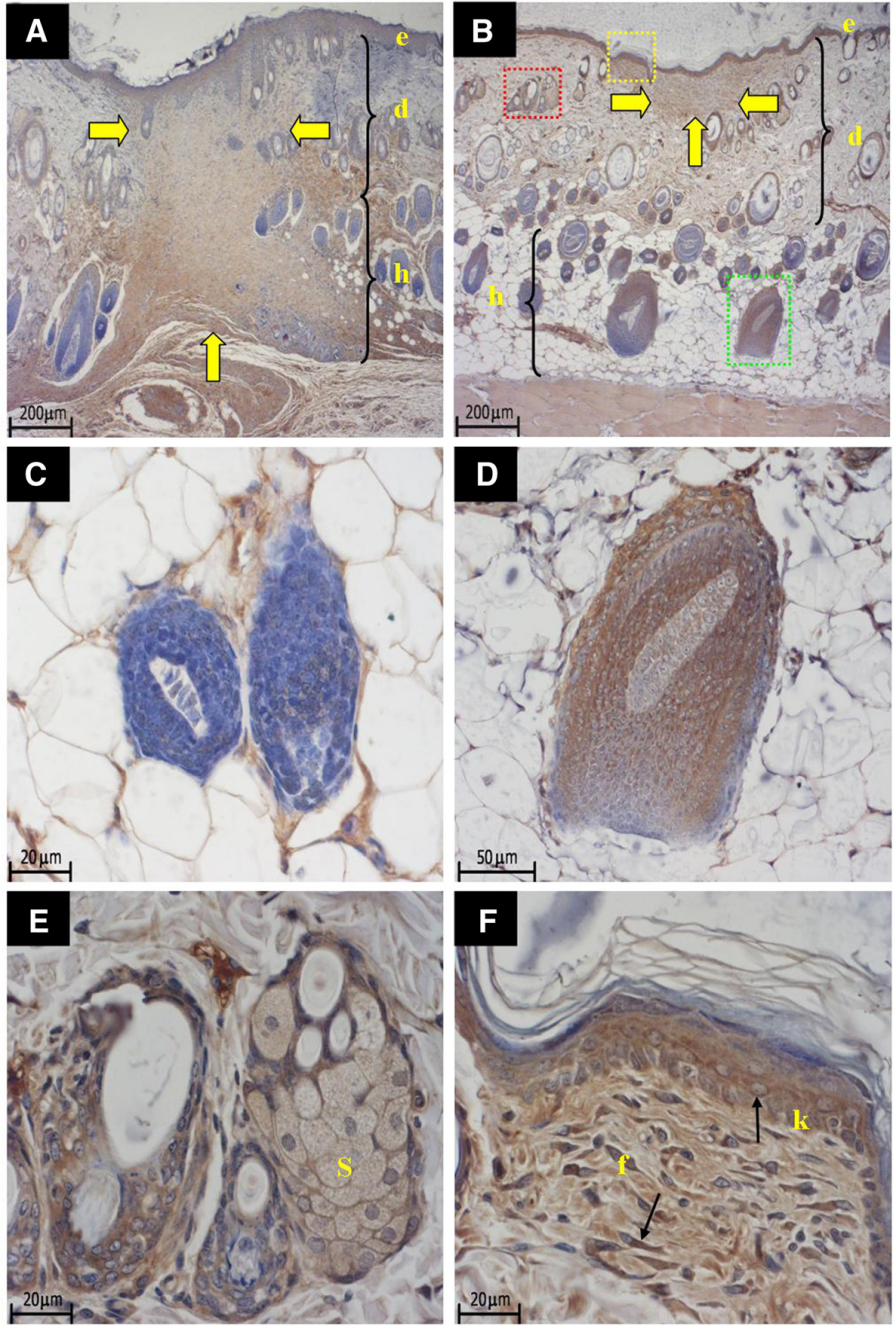
**A-B. Sections from the dorsal skin of the Ovx, and the Ovx+YCJ groups, following treatment for 14 days.** Sections were immunostained with anti-ER-α antibody. The yellow arrows indicate wound areas. Immunoreactivity is detected in keratinocytes, fibroblasts, sebaceous gland (red box), and hair follicle (green box). ***A *=** Ovx group; ***B *=** Ovx+YCJ group; ***e *=** epidermis; ***d *=** dermis; ***h *=** hypodermis. (4x). ***C-F*.** Higher magnification of B. ***C *=** non-immunoreactive hair follicles. (x40). ***D *=** Strong immunostaining of the hair follicle seen in the green box in B. (x20). ***E *=** Strong immunostaining of the sebaceous gland (S) seen in the red box in B. (x40). ***F *=** Strong immunostaining of keratinocytes (k) and fibroblasts (f) seen in the yellow box in B. (x40).

**Figure 5:**
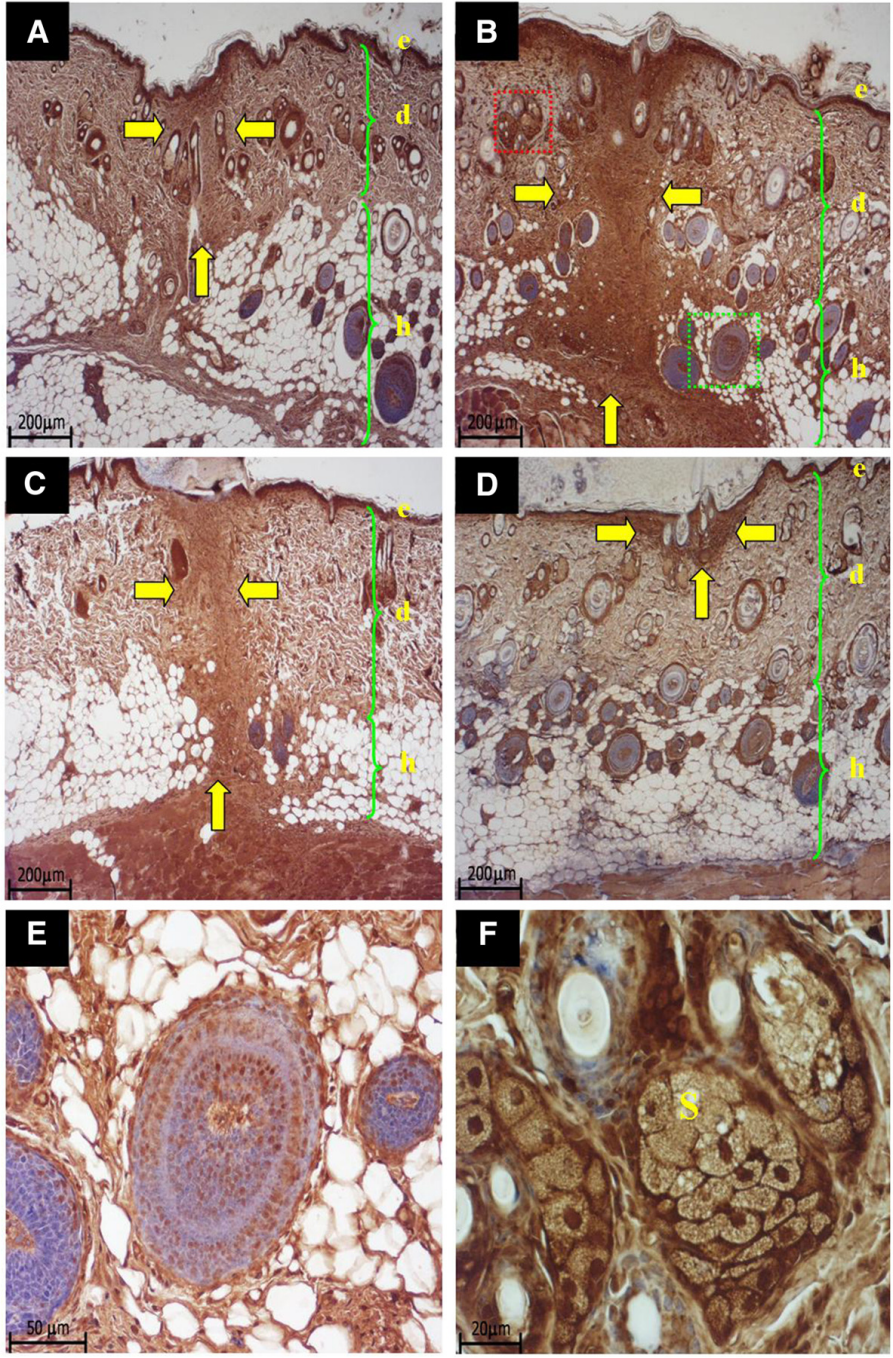
**A-F. Sections from the dorsal skin of the four groups, following treatment for 14 days.** Sections were immunostained with anti-ER-β antibody. Wound sites in A, B, C, D are indicated by yellow arrows. (x4). ***A *=** sham-operated group, ***B *=** Ovx group , ***C *=** Ovx+EB group, ***D *=** Ovx+YCJ group. **E.** Higher magnification (x20) of the green boxed area seen in B and showing strong ER-β positive immunostaining of the hair follicle. **F.** Higher magnification (x40) of the red boxed area seen in B and showing strong ER-β positive immunostaining of the sebaceous glands. ***e *=** epidermis, ***d *=** dermis, ***h *=** hypodermis; ***S *=** sebaceous glands.

The dermal wound depth and width were the smallest in the ovx+YCJ group, as compared to the other groups following a 14-day-treatment period (p < 0.01). This was associated with higher number and thicker dermal collagen fibers, as well as with significantly (p < 0.01) thicker epidermis (Figures 2A-G). The amount and size of collagen fibers of the ovx+YCJ group were similar to the control groups (sham, and ovx+EB group) (p > 0.05). The diameter of the hair follicles was significantly (p < 0.01) higher in the ovx+YCJ group, as compared to the other groups following the 14-day-treatment period (Figure 3A). Moreover, the number of these follicles, which were strongly immunostained against ER-α and ER-β, was significantly (p < 0.01) higher in the ovx+YCJ group, as compared to the other groups (Figures 3B, C, and Figures 4 and 5). Strong immunostaining against ER-α and ER-β was also observed in the keratinocytes, fibroblasts, and sebaceous gland in the ovx+YCJ group, as compared to the other groups (Figures 4 and 5). Finally, the results of the correlation analysis between dermal and hypodermal hair follicle thickness and serum E2 level were R^2^ -0.14 and -0.17, respectively.

## Discussion

For centuries, YCJ has been used for its various therapeutic effects, but not for wound healing. We were the first research group to report back in 2006 that ovx rats receiving YCJ at 100 mL/kgBW had significantly better wound healing, including less scarring, brighter skin, and softer hair, as compared to controls [10]. However, these observations were only based on gross morphological evaluation at that time. To take such observations one step further, we conducted the current study aiming at investigating at the microscopic level the changes taking place inside the wound following the intake of rats of YCJ over a seven and 14 days period. Moreover, the phytoestrogenic property of YCJ, as well as the possible role of such property in quicker wound healing was explored.

Our results demonstrated that the intake of YCJ over one week was not enough to produce significant microscopic healing changes in the wound area, as compared to controls. However, such changes were prominent when the intake continued for one more week. This was evident in the accelerated wound healing that was characterized by reduced wound depth and width, increased thickness of the epidermis and dermis, thicker and more abundant collagen fibers and hair follicles, and density of immunostaining against ER-α, and ER-β in the epidermis, dermis, and hypodermis. Such results seem to provide evidence to the observation that YCJ has phytoestrogenic properties, and that the latter could both act as SERM and play a role in the homeostasis of the epidermis, dermis, and hypodermis. This is supported by our recent experiment in which we used gas chromatography-mass spectrometry to confirm that the phytoestrogens of YCJ were sitosterol, stigmasterol, and campesterol [11]. This is also supported by other studies showing a stronger positive *in vitro* stimulatory effect of a SERM, Raloxifene, on collagen biosynthesis by human skin fibroblasts, as compared to traditional estrogen treatment [12]. This is also supported by the fact that collagen biosynthesis and deposition are an essential aspect of successful wound healing. Fotsis and colleagues reported that Genistein, one of the known isoflavones which have been shown to interact with animal and human estrogen receptors, was able to significantly reduce the degradation of the extracellular matrix in the skin [13]. Pirilä and co-workers investigated the effects of estrogen and a potent matrix metalloproteinase inhibitor, chemically modified non-antimicrobial tetracycline, CMT-8, on wound healing in ovx rats [14]. They observed that estrogen can promote wound healing in ovx rats by normalizing wound bed total collagen structure and content, and by recovering the processing and expression of essential molecules in wound healing, such as laminin-5 gamma2-chain [14]. Pirilä and co-workers also demonstrated in a separate study that estrogen has a beneficial effect on skin wound healing in ovx rats by increasing the collagen content, and by reducing the matrix metalloproteinases (MMP)-mediated collagenolysis [15]. Shuster and co-workers reported a direct relationship between skin collagen and dermal thickness in a study correlating age and sex with skin thickness and collagen density [16]. Azzi and colleagues described specific effects of sex steroids on skin morphology [17].

Both estrogen and SERMs act through binding to homodimeric and heterodimeric ERs. SERMs, in addition, have the potential to selectively induce ER isoforms in a specific target tissue, thus amplifying further the effects on down-stream gene expression. Estrogen acts via ER, and there are currently two known estrogen receptors, denoted ER-α and ER-β. ER-α is expressed in the skin of both humans and rodents, whereas conflicting results have been reported regarding the expression of ER-β in the skin [18-20]. Therefore, we examined in this study the effect of YCJ intake on the expression of ER-α and ER-β in the wounded and normal skin, exploring the possibility that it has phytoestrogenic properties, and that it could act as SERM. Our results demonstrated both ER-α and ER-β were detected in the epidermis, dermis, hypodermis, hair follicles, sebaceous glands, keratinocytes, fat cells, fibroblasts, and panniculus carnosus (skeletal muscles) of the normal and wounded skin. Interestingly, the expression of ER-α and ER-β was significantly the highest in the group which received YCJ following the excision of the ovaries. Such expression was noted in both the nucleus and the cytoplasm. Hart and colleagues reported that the acute effects of estrogen on neuronal signaling were most likely mediated by extra-nuclear estrogen receptors associated with the plasma membrane and/or cytoplasmic organelles [21]. Thornton and co-workers demonstrated that ER-β was strongly expressed in the nucleus, while ER-α was present in diffuse cytoplasmic granules in human dermal papilla cells [22].

Our results showed that the number of hair follicles and their diameter either in the dermis or in the hypodermis of the ovx+YCJ group was the highest and largest, respectively, while the dermis and hypodermis of the ovx+EB group contained the lowest number of follicles and their diameter was the smallest. In contrast, we observed a strong negative correlation between the serum E2 level and the number and size of the hair follicles in the ovx+YCJ and ovx+EB groups. These findings indicate that YCJ had an agonistic effect on hair follicles, while exogenous estrogen (EB) has an antagonist effect. In agreement with our findings, E2 was found to effectively block hair growth in female mice [20]. In humans, E2 was found to be locally produced by the hair follicles [23]. Furthermore, a slower rate of replacement of spontaneous hair loss or plucked hair has been observed in pregnant women, an effect possibly related to the high levels of circulating estrogens [24].

The importance of hair follicles in skin biology does not rest solely with its ability to produce hair. Hair follicles are self-renewing, contain reservoirs of multipotent stem cells that are capable of regenerating the epidermis, and, accordingly, are thought to be actively involved in accelerating wound healing [25,26]. The present study supports this theory.

## Conclusion

In summary, we are reporting some novel findings in relation to the effects YCJ on cutaneous wound healing. YCJ does not only increase epidermal and dermal thicknesses, but also increases the number as well as the size of hair follicles and it restores collagen fiber size. Another beneficial effect of YCJ is the reduction of hypodermal thickness. One might argue that the reduction of hypodermal thickness may indicate reduced fat tissue leading to thinning of the skin and, thus, accelerating appearance of age wrinkles. However, the appearance of age wrinkles does not solely depend on the amount of adipose tissue present, but also, and in a more significant manner, on the amount and thickness of collagen fibers. It was clear in the photos presented in this study that the amount and size of collagen fibers of the ovx+YCJ group were similar to the control groups (sham, and ovx+EB group). Moreover, our lab has conducted a separate study, which focused on examining and quantifying collagen and elastic fibers using electron microscopy and image analysis. The data obtained confirm the results and argument presented in the current study, and will be presented in a separate manuscript due to the large amount of photos included in the current study.

The encouraging findings presented in this study could have some exciting clinical implications, and could highlight the importance of conducting a clinical trial on the effects of the use of YCJ to treat chronically-delayed cutaneous ulcer healing in the elderly.

## Competing interest

The authors hereby declare that there is no conflict of interest in this manuscript.

## Authors’ contributions

NR: Experimental design, conduction of animal study, and analysis of data, and drafting the initial manuscript. FS: Histological analysis, analysis and interpretation of data, presentation of photos and figures, re-writing the manuscript, and critically revising the manuscript for important intellectual content. IS: Participating in the experimental animal work. KS: Participating in the analysis of YCJ. PS: Immunohistochemistry. PB: Image analysis. WR: Participating in the analysis of YCJ. WM: Participating in the histological analysis. All authors read and approved the final manuscript.

## Pre-publication history

The pre-publication history for this paper can be accessed here:

http://www.biomedcentral.com/1472-6882/12/252/prepub

## Supplementary Material

Additional file 1 **Figure S1.** Wound following a seven day treatment period. Wound following a seven day treatment period. *A* = sham-operated group, *B* = Ovx group , *C* = Ovx+EB group, *D* = Ovx+YCJ group. Click here for file

Additional file 2 **Figure S2.** Wound following 14 days treatment period. Wound following 14 days treatment period. *A* = sham-operated group, *B* = Ovx group , *C* = Ovx+EB group, *D* = Ovx+YCJ group. Click here for file
