# Supplementary figures and images for: Young coconut juice can accelerate the healing process of cutaneous wounds
Source: BMC Complement Altern Med. 2012 Dec 12;12:252. doi: 10.1186/1472-6882-12-252 (PMC3538627; doi:10.1186/1472-6882-12-252)

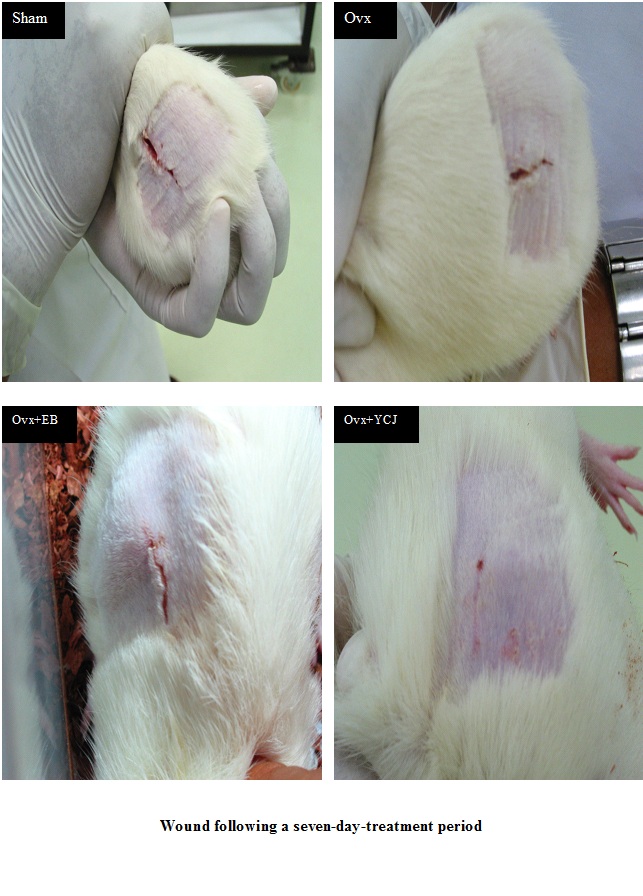

Supplement: Additional file 1 — Figure S1. Wound following a seven day treatment period. Wound following a seven day treatment period. A = sham-operated group, B = Ovx group , C = Ovx+EB group, D = Ovx+YCJ group. [file 1472-6882-12-252-S1.jpeg]

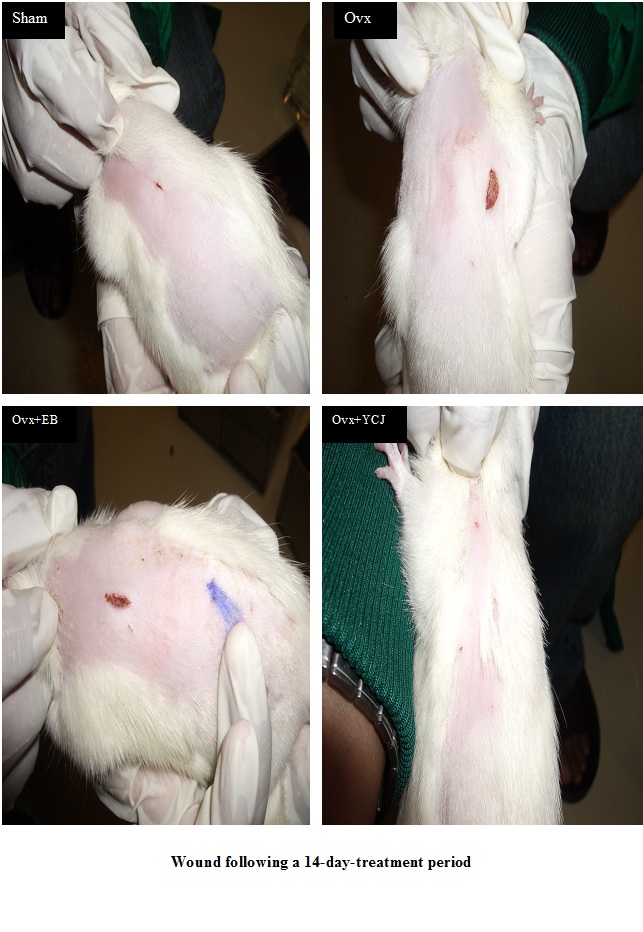

Supplement: Additional file 2 — Figure S2. Wound following 14 days treatment period. Wound following 14 days treatment period. A = sham-operated group, B = Ovx group , C = Ovx+EB group, D = Ovx+YCJ group. [file 1472-6882-12-252-S2.jpeg]
